# Microbial Degradation of Pesticide Residues and an Emphasis on the Degradation of Cypermethrin and 3-phenoxy Benzoic Acid: A Review

**DOI:** 10.3390/molecules23092313

**Published:** 2018-09-11

**Authors:** Yichen Huang, Lijuan Xiao, Feiyu Li, Mengshi Xiao, Derong Lin, Xiaomei Long, Zhijun Wu

**Affiliations:** 1 College of Food Science, Sichuan Agricultural University, Ya’an 625014, China; 18723091689@163.com (Y.H.); 18728194686@163.com (L.X.); 18728190420@126.com (F.L.); 18227591863@163.com (M.X.); 18428301943@126.com (X.L.); 2 College of Mechanical and Electrical Engineering, Sichuan Agricultural University, Ya’an, 625014, China; wzj@sicau.edu.cn

**Keywords:** biodegradation, characteristic, degradation mechanism, degrading bacteria, pyrethroid

## Abstract

Nowadays, pesticides are widely used in preventing and controlling the diseases and pests of crop, but at the same time pesticide residues have brought serious harm to human’s health and the environment. It is an important subject to study microbial degradation of pesticides in soil environment in the field of internationally environmental restoration science and technology. This paper summarized the microbial species in the environment, the study of herbicide and pesticides degrading bacteria and the mechanism and application of pesticide microbial degrading bacteria. Cypermethrin and other pyrethroid pesticides were used widely currently, while they were difficult to be degraded in the natural conditions, and an intermediate metabolite, 3-phenoxy benzoic acid would be produced in the degradation process, causing the secondary pollution of agricultural products and a series of problems. Taking it above as an example, the paper paid attention to the degradation process of microorganism under natural conditions and factors affecting the microbial degradation of pesticide. In addition, the developed trend of the research on microbial degradation of pesticide and some obvious problems that need further solution were put forward.

## 1. Introduction

From the end of the 20th century to the present, the total global grain output has increased from 500 million tons to 700 million tons now [1]. Among them, cereals account for 80% of human consumption of food [2]. Food is endangered by pests during its natural growth or storage. For example, China is a largely agricultural country, but 40 million tons which is about 8.8% of the country’s total grain output are lost in vain because of a variety of insect pests every year [3]. India produces an average of 250 million tons of grain a year, but it also loses 11–15% of its total output, or about 27.5–37.5 million tons a year, due to pests and other causes [4]. To avoid such losses, pesticides are widely used to control agricultural and household pests [5]. The loss of food has been reduced a lot after the use of pesticides, but these pesticides are widely distributed in the soil, water, air, and agricultural products. So, the wide use of pesticides causes a great potential threat to the environment [6,7]. They not only pollute the soil and crops, but also further pollute the ground water as well as the marine environment, which directly threatens human’s health and environment [8,9,10,11,12,13].

In order to solve the contradiction between agricultural products with high yield or stable production and environmental pollution, we can start from the following two aspects. On the one hand, pesticides with low toxicity, high efficiency and low pesticide residues should be found and developed, on the other hand, ways of degrading pesticide residues also should be fundamentally worthy of attention. Studies on microbial degradation of pesticide residues originated in 1940s, and as people pay more attention to the environment, the research on the degradation process and degradation mechanism of organic pollutants has been deeply studied. [14]. Bacteria in nature could degrade the pesticide residues, with low cost and environmentally friendly and it would not cause secondary pollution. But the efficiency was relatively slow and the natural environment was complex and changeable, which may affect the feasibility and efficiency of microbial degradation of pesticides. Consequently, researchers have conducted fine studies of bacteria and had a clear understanding of the degradation mechanism of organic pesticides. Among them, a number of bacteria that could degrade and convert pesticides have been isolated [15,16,17]. Besides, the mainly degradable ways and mechanisms of pesticides were described clearly [16,18,19,20]. According to the researches, the current studies of biodegradable pesticides that are mainly concentrated in the microorganism in the soil, such as fungi, bacteria, and actinomycetes [21], of which the main role are bacteria and fungi. Because the bacteria could easily induce mutant strains, which also had a variety of biochemical capacity to adaptive environment, and thus more in-depth study can be carried out [22,23,24,25,26].

The review can not only provide massive data and information for further study on the biodegradation of cypermethrin (CY) pesticides, in which were the detection method, model for screening microbes, degradation characteristics, and degradation pathway and mechanism, but also enriched and perfected the theory of biodegradation of CY pesticides. In addition, this review analyzed the degradation process of 3-PBA. The microbial degradation of CY and 3-PBA was studied, a new way for the breeding of degrading bacteria was provided, and the resources of degradable bacteria were enriched. Therefore, the review was of significant meaning and value to provide reference for other studies, such as biodegradation of other types of pesticides or veterinary drugs, using CY pesticides to biodegrade environmental pollution, eliminating or reducing pesticide residues in agricultural products.

## 2. Research on the Progress of Microbial-Degradation of Pesticide Residues

### 2.1. The Main Types of Pesticides in Agriculture

Organochlorine pesticides were very stable in the environment, and were easily enriched in the organisms’ and human’s body through the food, so China had forbidden producing and using them from 1983 onwards, such as hexachlorocyclohexane (666), 1,1,1-trichloro-2,2-bis(p-chlorophenyl) ethane (DDT) and other organochlorine pesticides [27,28], in which like methamidophos in organic phosphorus were gradually phased out. Several types of organic pesticides were used in agriculture as shown in Table 1.

In nature, there were a large number of microorganisms with strong adaptability and different types of metabolic. They could use a variety of synthetic organic matters like lucerne or horn meal, organophosphonates as carbon source, nitrogen source and energy. This would be conducive to their growth [5,50,51], and also could completely mineralize or degrade the organic pesticide into small non-toxic molecules through various metabolism ways, and then ultimately achieved the purpose of purifying the environment [45].

### 2.2. Types of Pesticides-Degrading Microorganism

In recent years, many scientists have enriched, isolated, cultured and screened a lot of microbial floras, such as bacteria, fungi, actinomycetes, algae and other microbial strains from the natural sewage or soil to degrade pesticides. Kafilzadeh et al. [52] separated bacteria from sediments and water samples from high agricultural activity areas for the detection of endosulfan degradation. It was found that the five bacteria genus klebsiella, acinetobacter, alcaligenes, flavobacterium, and bacillus could degrade endosulfan effectively. Jayabarath et al. [53] selected 319 actinomycetes from saline soils of Sangli District (Maharashtra) for carbofuran tolerance test, while only the seven strains of Streptomyces alanosinicus, Streptoverticillium album, Nocardia farcinia, Streptomyces atratus, Nocardia vaccini, Nocardia amarae, and Micromonospora chalcea can grow and degrade pesticides very well. Elgueta et al. [54] used white-rot fungi to study the degradation of atrazine and found that the half-life of atrazine decreased to six days. Besides, Kabra et al. [55] studied the ability of green microalga Chlamydomonas mexicana to degrade atrazine and found that microalgae could effectively degrade atrazine by accumulating atrazine in cells and then degrading it, reaching a degradation rate of 14–36%. Among them, the isolated bacteria were mainly Pseudomonas, Klebsiella pneumoniae, Bacillus subtilis, etc. Fungi included mycobacterium, Aspergillus, white rot fungi, etc. Algae included marine chlorella, etc. The common pesticide-degrading microorganisms were listed, as shown in Table 2.

### 2.3. Mechanism of Microbial Degradation of Pesticides

Pesticides in the soil could be degraded by different ways; the traditional methods included physical degradation, chemical degradation, and physical-chemical degradation, which basically caused secondary pollution [67,68,69]. In recent years, microbial degradation was used more frequently because pesticides were used as mainly microbial nutrient, and ultimately decomposed into some small molecules, such as CO_2_ and H_2_O. The progress was called enzymatic reaction, which included that the compound got into microorganism’ body through a certain way firstly, and then through a series of physiological and biochemical reactions under the action of various enzymes, finally pesticide would be completely degraded or broken down into smaller molecular compounds which have non-toxicity or less toxicity [45,70]. For example, Pseudomonas sp strain ADP used atrazine as the only carbon source, and three enzymes were involved in the first few steps of degradation of atrazine. The first enzyme was AtzA, which catalyzed the reaction of hydrolysis dechlorination of atrazine to non-toxic hydroxyl atrazine, and it was a key enzyme of atrazine’s biological degradation. The second enzyme was AtzB, which catalyzed the dehydrochlorination of the hydroxy atrazine to produce N-isopropyl cyanuric amide. The third enzyme was AtzC, which catalyzed the cyanuric acid and isopropylamine formated by N-isopropyl cyanuric amide. Finally, atrazine was degraded to CO_2_ and NH_3_ [71,72]. As degrading enzymes were often more resistant to abnormally environmental conditions than microbial cells that could produce such enzymes, and the degradable efficiency of enzymes was much higher than that of microorganism, especially for low concentrations of pesticides. Therefore, that people wanted to utilize degrading enzymes to purify the environment, which was polluted by pesticides would be a more effective way. However, the degrading enzyme was easily inactivated under the effect of non-degeneration and soil adsorption in the soil, so it was difficult to maintain the degradable activity for a long time. Also, the poor mobility of the enzyme in the soil and other factors limited the application of degrading enzymes in practice [22,23]. Many trials have been demonstrated that most of the genes encoding these enzymes were controlled on the plasmid [9,46,73], for example, the bio-degradation of 2,4-D was controlled by the gene carried on the plasmid [74]. Pesticides were degraded through the expression of plasmid gene and chromosome gene in the bacteria.

The degradable ways included oxidation (hydroxylation reactions, such as aromatic hydroxylation, aliphatic hydroxylation, N-hydroxylation, epoxidation, N-oxidation, P-oxidation, S-oxidation, oxidative dealkylation, oxidative dehalogenation, and oxidative deamination), reduction (reduction of nitro group, quinone reduction, and reductive dehalogenation), hydrolysis (some esters such as thiophosphate, thiocarbamate, etc., which have ester bonds that can be hydrolyzed by bacteria), dehydrogenation, dehalogenation, decarboxylation, condensation, synthesis and so on [35,42]. The bacteria would convert organic macromolecules into small non-toxic molecules, thus avoiding the secondary pollution. Studies have shown that mineralization and co-metabolism were the main mechanisms for the further degradation of pesticides and their intermediate products [18,75,76,77].

The whole degradation mechanism was divided into three parts. Firstly, adsorption of target, it took place on the surface of the cell membrane and was a dynamic equilibrium process that was also critical. Secondly, the target got into the cell through the surface of the cell membrane, and the penetrated rate and efficiency were related to the molecular structure of the target isomerism. Thirdly, xenobiotic target conducted rapidly enzymatic reaction in the membrane [78].

**Figure 1:**
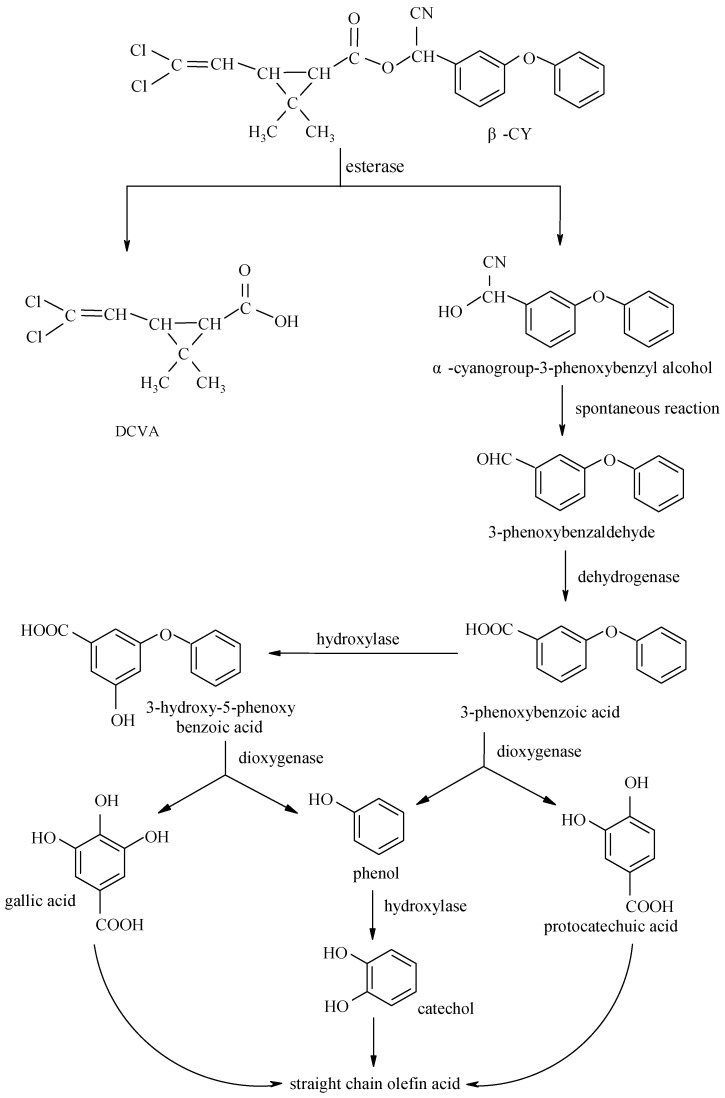
Degradation pathway of beta-CY (β-CY) by Aspergillus niger YAT (Taken from Deng et al. 2015).

Mineralization was a general term for the conversion of organic compounds into inorganic compounds under the action of soil microbes. Many chemical pesticides were analogs of natural compounds, and some microorganism had the enzymes to degrade them. They could be used as a source of microbial nutrients and then be degraded to inorganic matters, carbon dioxide, and water by microorganism. Mineralization was an ideal way to degrade because pesticides were completely degraded into non-toxic inorganic substance. Co-metabolic referred to that some chemical substances like insecticides, fungicides, and herbicides, etc. which did not exist in natural conditions, could be degraded not by bacteria or fungi easily, but only by adding some organic matter such as exogenous or iso-biomass as the primary energy [79]. Taking a type of co-metabolism as an example, the degradation products of the monomethylamine products of Pseudomonas dendrolimus DR-8 were 2,4-dimethylaniline and NH3, whereas the DR-8 strain could grow with other organic nutrient substrates added as carbon source and energy source instead of meth amidine, meanwhile the degradable products were not completely mineralized [80]. Co-metabolism played a major role in the microbial degradation of pesticides. It should be noted that in most cases, the synergistic effect of a series of reactions rather than a single reaction was needed to complete the degradable process of pesticides in the microbial body. For example, Deng et al. found that Aspergillus niger YAT could degrade beta-CY(β-CY) and its intermediates completely by co-metabolism and mineralization way, and the whole degradable process was analyzed, while there was rare analysis in other pyrethroid degrading strains. The degradable pathway of β-CY by Aspergillus niger YAT could be seen in Figure 1 [81].

### 2.4. Factors Affecting Microbial Degradation of Pesticide Residues

Microbial degradation of pesticide residues was restricted by many factors which was divided into internal factors and external environmental factors, in which the effect of internal factors originated from the structure of pesticide and the micro-organisms.

(1) Degradation and transformation of pesticides was affected by microbial species, metabolic activity, and adaptability directly [82].

Many experiments have shown that the reactions of different species of microorganism or the same species of different strains to the same organic substrate or toxic metal were different, and the microorganism had a strong ability to adapt environment and to be domesticated. Through the adapted process, the new compounds could induce microorganism to produce the corresponding enzyme system or establish a new enzyme system to degrade them. Functional characteristics and changes of degradation were the most important factors [83,84,85,86].

(2) Effect of pesticide structure

Pesticides’ own factors, such as their molecular weight, spatial structure, the number and type of substituents, substituted characteristics and location affected the rate and efficiency of microbial degradation of pesticides [87,88,89]. In general, the polymer compound was less biodegradable than the low molecular weight compound. The polymer and composite were more resistant to bio-degradation, but that with simpler space structure was degraded more easily [90]. Microbial degradation of rhizosphere was the main route of phytoremediation on soil contaminated by polycyclic aromatic hydrocarbons (PAHs). Plant absorption was a relatively minor pathway, and mixed planting could improve the efficiency of these two ways simultaneously. For different kinds of PAHs, plants were easier to absorb 2–4 ring PAHs.

The use of herbicides has become an indispensable means of agricultural production, and thus many problems of environmental pollution have become increasingly prominent, such as the threat to the living environment and excessive pesticide content of agricultural and sideline products. Then, contaminated agricultural products got into human’s body and harm human’s health by the bio-accumulation of food chain and so on. Most of the current contaminants were synthesized biological heterologous organic substances which did not exist in nature, they often showed a strong resistance on the degradation of microorganism. It may be explained that the time of these compounds’ getting into the nature was relatively short so that single microorganism has not evolved the metabolic mechanisms about degradation of such compounds. Although some dangerous compounds may be degraded slowly in nature through the mineralization and co-metabolism by natural formed microbial populations, this was still a new challenge for the microbial world. The process of microbial degradation was very slow, and it may need to change some structure. When comparing with the currently widely used synthetic bio heterologous substances, the natural evolutionary process of microorganism was clearly unable to meet the requirements of microbial pesticides’ degradation, as the speed of the process was far from reaching what the environment and human needed. Thus, the balance of the entire ecosystem would be destroyed after a long-term effect [18]. Therefore, it was very important and urgent to study some of the methods that can make microbial flora achieve maximum degradation of pesticide in a relatively short time.

(3) Environmental factors

Temperature, humidity, salinity, pH, nutrition, carbon dioxide, oxygen, substrate concentration, surfactant, etc. would affect the degradation [91,92,93,94]. Bacteria or their enzymes needed a suitable temperature, pH and substrate concentration [95]. The number of benzene rings of PAHs had a great impact on the microbial degradation of PAHs. The degradation of two rings and tricyclic compounds (naphthalene, phenanthrene, anthracene, fluorene, etc.), which existed in the environment only need a short time, and microorganism can mineralize these compounds with using PAHs as the sole carbon source. However, the four-ring and other multi-ring PAHs with high molecular weight were stable in the environment so that they were difficult to be degraded. But, the white rot fungi could degrade these compounds by metabolism [96]. Generally, with the increase of the number of benzene rings of PAHs, octanol/water partition coefficient increased, and the degradable rate was lower and lower. Surfactant could change the solubility of PAHs in soils, the balance of adsorption and desorption, and the interaction between PAHs and soil microorganism, thus further chang the bioavailability of PAHs. For example, Yuan et al. used the way of reducing the interfacial tension between soil and water to increase the solubility of PAHs, facilitate the transport of PAHs, and thus the bioavailability of PAHs increased. However, due to the toxic effects of surfactants on microorganism or the use of non-toxic surfactants as microbial growth matrix, the bioavailability of PAHs might be inhibited. In addition, the effect of surfactants on the bioavailability of different forms of PAHs in soils was different, so that the surfactant could be added to increase the solubility of PAHs in the aqueous phase, promote solid phase transferring to the water phase, improve bioavailability, and reduce the surface and interfacial tension of matrix [97].

The lack of nutrients was an important limiting factor for microbial growth and maintenance of population. Lewis and other studies have shown that maintaining the normal ratio of C:N:P in the polluted environment can promote the degradation of PAHs stably. In order to have a complete degradation and to speed up the purification rate, ammonia and phosphate were often added to adjust the C: N: P ratio in bio-repair.

Temperature and humidity were the most important factors, which affected the growth and reproduction of bacteria [98]. Zhu et al. investigated that the degradation and mineralization of biaryl compounds in soil and compost by bacteria Ralstonia and Pickettii, and found that the nonionic surfactants tween 80 can enhance bacteria’s utilization of biaryl compounds under suitable soil moisture conditions, such as biphenyl, 4-chlorobipheny [99,100]. Gupta et al. thought that the effect of organic substrate content on pesticide’s degradation in composting was greater than that of bacteria content when compost was mixed with soil contaminated by PAHs. Because bacteria did not produce energy and need other carbon and energy source, so nutrition was more important when bacteria degraded pesticides by co-metabolism [101,102].

At present, pesticides in agriculture were mainly including organic phosphorus, organic chlorine, carbamate, pyrethroid, chloronicotinyl insecticide and some other fungicide, etc. Many microorganisms that degraded pesticides could be screened from natural sewage or soil, which included bacteria, fungi, actinomycetes, algae, and other microbial strains. Bacteria were Pseudomonas, Klebsiella sp., Bacillus subtilis, etc. Fungi were Trichoderma spp., Aspergillus spp., white rot fungi, etc. Algae had marine chlorella, and so on. In addition to the traditional methods, such as physical degradation method, chemical degradation method, and so on, the microbial degradation method was commonly used in pesticide degradation. This method had high efficiency, low cost, and good degradation effect. Microorganism used some substances in pesticides as nutrients and decompose them into small molecules, and the main ways of degradation were mineralization and co-metabolism. The effects of degradation were influenced by many factors, such as the type of pesticide, the type of microorganism, and temperature, humidity, acidity, and air’s composition in the environment. The purpose of these studies was to screen the most suitable microorganism for different pesticides, the most suitable degradation methods and degradation environment, which provided a more convenient reference for future research.

## 3. Present Situation of Degrading Pesticides by Micro-Organisms

At present, there were many researches that were devoted to microbial degradation of pesticides [37,103,104,105]. For example, there was a type of biological technology named the immobilized bacteria technology rising in the just 1980s, namely using free cells or enzymes fixed in the limited space, and keeping them active meanwhile, which also can be reused [106,107,108]. This technology was characterized by efficient use of two strains with low pollution.

Construction of the system of several bacteria allowed for several bacteria to solve the problem of the incomplete transformation of single strain [24,25]. A study discussed the microbial cell’s surface display technology that developed in the middle of 1980s [109,110], which showed a process about the combination of exogenous protein (this protein has enzyme activity) with degradable strain, this procedure was active and it could combine the function of transport and secretory so as to make the exogenous protein express better. Finally, the exogenous protein was embedded in the surface of cell membrane and played the specific function of the customary exogenous proteins. This method created a direct contact between the bacteria and the pesticide residues, which not only simplified the purification process of proteins, but also improved the degradable rate [111,112].

In addition, in 1994, American scientist Stemmer et al. introduced the advanced technology of simulated DNA shuffling in vitro contained in Darwinian theory. The reorganization technology made the activity of enzymes greatly improved, and it did not require the three-dimensional structure determination of enzyme [113]. People have done a lot of researches on the degradable plasmid of the herbicide 2,4-D and 2,4,5-T, and these studies proved that the main 2,4-D-degrading bacteria were Peseudomonas sp. and Alicaligenes sp. which contained the plasmid of pjP4 Alicaligenes eutophus JMP134d [22,23,97].

Ma et al. [114] used Southern hybridization and plasmid curing to get the fact that the naphthalene dioxygenase (ndo) gene of PAH degrading bacteria Pseudomonas sp. would be immobilized on a large self-transmissible plasmid and then transferred to thermophilic strains, and similar temperature optim was also observed. The study on plasmid all over the world in the separation and screening of degrading bacteria strains were of great deal, but there was very few on the degradable bacteria. Most of them were applied degrading bacteria directly in the pot experiment.

The results of a number of researches showed that the degrading bacteria isolated from both pot experiment and field experiment had good degradation, and the degradable rate even achieved more than 70%. The degradable rate of most strains was more than 90%, which greatly shortened the half-life.

Some studies have shown that certain enzyme could endure the variation of environmental conditions while the bacteria which produced this enzyme could not. For example, Parathion Hydrolase could tolerate salt’s concentration as high as 10%, 1% of the solvent concentration and 50 °C of the temperature, but Pseudomonas which could produce this enzyme in this condition cannot grow. The immobilized enzyme not only had the good purified effect of CY, but also degraded organophosphorus and pyrethroid pesticides.

Microbial degradation of pesticide pollution by using composite systems with a variety of microbial taxa was an inevitable trend, which was close coordination between the composite nature of bacteria and comply with the laws of nature. The artificially composite systems of micro-organisms were inoculated to pesticide pollution in soil or to improve the utilization of agriculturally organic waste composting have been a good way to deal with polluted soil. The modern city life’s garbage, organic solid waste, sewage sludge contains large amounts of organic pollutants and heavy metals, and agriculturally organic solid wastes all contained a large number of pesticide residues and other pollutants. In the process of composting, the pollutants were eliminated by microbial degradation and volatilization, leaching, photolysis, chelation, complexation, and so on [115]. At the same time, the microbial system was compositely contained in the active compost, and it was more likely to become the dominant flora of polluted soils. Therefore, the composting method could not only eliminate pollution, but also get high quality compost products, which had great significance on sustainable development for environmental pollution control and agricultural major.

The author deemed that the construction of cooperative relations between strains could not only improve the efficiency of lignocellulose decomposition composite system, but also improve the long-term stability of constituent species, and it was not easy to be contaminated [14,116]. On this basis, the complex system gave the function of pesticide decomposition, which had a strong ability to decompose a variety of pesticides and it had a well applicable effect.

In short, although the microbial degradation of pesticide residues had made initial progress and most kinds of microbial strains to degrade pesticide had been identified, the practical application of microbial bioremediation was often affected due to the degradation efficiency was low. So, the microbial degradation of pesticide residues was still a problem that needed to be overcome.

## 4. Microbial Degradation of CY and its Application

### 4.1. Overview of CY

Pyrethroid insecticides were synthetic pesticides used to kill warms that were based on natural pyrethrum structure. At present, the amount of pyrethroid, organophosphate, and carbamate was accounting for about 20% of the world market [117]. The acute toxicity of pyrethroid pesticides was smaller and the doses were lower. In the past, the pyrethroids have been considered to be degraded by oxidase system in vivo and it does not have accumulation. In other words, these pesticides were safe so they get wide use, and the amount of pesticides has been increasing gradually in the production of fruits and vegetables. However, the recent studies have shown that these pesticides had accumulated in animal and plant bodies, and they may cause chronic disease in long-term exposure [118]. Some types of pyrethroid pesticides had carcinogenic, teratogenic, and mutagenic effect [119]. In a way, the pesticides were provided with moderate neurotoxic effects, immune system, cardiovascular toxicity and genetic toxicity on mammalian toxicity and there was no specific drug to treat the toxicity [120]. Therefore, all the countries in the world had made a limited standard for pyrethroid pesticide residues in the products, and the excessive pesticide residues was one of the most important obstacles to export agricultural products.

### 4.2. The Structure and Overview of CY

CY was a commonly used pyrethroid pesticides, molecular formula C_22_H_19_C_l2_NO_3_, molecular weight of 416.32. There were some functional groups in the CY molecule, such as ester bond, halogen double bond, cyanide bond, and benzyl carbon. There were three asymmetric carbon atoms, one was combined with cyano group, and the others were the first and the third carbon atoms of the cyclopropane carboxylic acid. Therefore, CY had four raceme and eight isomers, and CY was in the same side of the hydrogen atom with the first position of the alkyl group and the carbon atom of the third order, which was known as cis isomers, and in the opposite side was called trans isomer [121].

CY was one of the most commonly used pyrethroid pesticides and accounting for about 50% in the CY pesticide market [122]. The pesticides were produced in 1975 by the British Mitchell Cotts, ICI, FMC, Swiss Ciba-Geigy, Japan Sumitomo, and Dutch Shell, which could be used for the treatment of tea, vegetables, fruit trees, cotton flowers and trees, and the prevention and control of pests and parasitic disease. Otherwise, pesticides characteristics were seized to make the environment stable, slow the process of degradation, and reduce the degradable rate [123].

There were obvious differences between different isomers of CY in insecticidal effect and photolysis rate, the insecticidal activity from strong to weak was cis-, trans- and cis trans-CY [121], insecticidal activity of the ester was highest, which was made of the ring structure of the acid and S configuration of alcohol.

It played an important role in the prevention of pests in crop. With a number of highly toxic organophosphorus pesticides banned, the scope of application of CY was more and more widely, and its usage was increasing. But CY was not easy to be degraded by air and light, which made its half-life in the natural environment up to 94-1103 days.

### 4.3. Study on Degradation of CY at Home and Abroad

**Figure 2:**
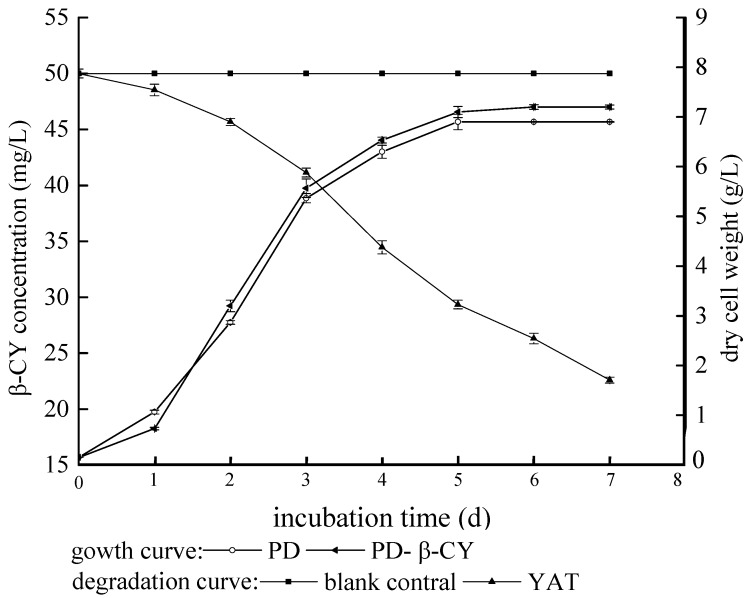
Growth characteristics and β-CY degradation curves of *Aspergillus niger* YAT (Taken from Deng et al. 2015).

At present, the degrading bacteria for CY degradation on the domestic and foreign were Micrococcus sp., Serratia sp, Klebsiella sp., Bacillusu sp., Rhodococcus sp., KlebMella sp., seudom onasaeruginosa, Aspergillus terreua, Monilochaetes and Fusarium, Alcaligenes sp., and so on. Deng et al. studied the kinetic parameters of the degradation of β-CY by YAT under the effects of different factors, and the results were shown on Table 3. With the substrate concentrations of 25–100 mg/L, the half-life of β-CY was rather varied and enlarged with the increase of substrate concentrations. The particular strain could degrade β-CY validly over a range of temperatures (25–35 °C) and pH (6.0–8.0), which made pesticide-degrading bacteria had advantages in the environment. Except that, they also discussed the growth’s characteristics and the degradable capacity of β-CY for Aspergillus niger YAT (Figure 2). The results showed that β-CY made no difference in the growth of YAT in PD, it prolonged YAT’s lag phase, but it did not affect its final amount of growth. Accompanying with rapid growth of YAT after 24 h, β-CY was degraded rapidly, degradable rate reached 41.32% at 120 h. Then strains basically did not grow, and the degradable rate reached 54.83% at 168 h [81]. Deng et al. [81] received Bacillus licheniformis that were isolated from enriched culture in the activated sludge, and used 10% CY with EC to spray vegetables. After one day, there were some experiments on the field sprayed degrading bacteria, and then after every five days collected sample. The results of gas chromatography analysis were that the bacteria could effectively remove CY residues in vegetable’s surface, and within five days of the removing rate was 64%. In the experiment, the degradable rate of CY in tea was up to 68.94%. *Enterobacter cloacap*, which was screened from the sludge in the sewer of pesticide’s factory, was dealt with 100 mg·L^−1^ CY 3D and the medium was fermented broth based on the environment of 30 °C and the pH of 7.0. At last, the degradable rate of CY was over 50% [124].

From the farm soil and the mixed soil composed of various soil samples, Serratia sp. and Pseudomonas aeruginosa were isolated, which could degrade CY at least 50% in 20 days [125]. Fusarium isolated from soil was cultured for eight days under the conditions of 28 °C in the rotary table, and then incubated for five to seven days in 26–30 °C. By high-performance liquid chromatography (HPLC) analysis of CY in 4 °C, 60–80% of the 100 mg/L CY could be degraded in 5 days [126]. By enrichment incubation, bacillus subtilis separated from the sludge were used to degrade beta- cypermethrin [127]. The efficiency of some bacteria to degrade CY was constrained by environmental minerals, carbon sources, water and so on [125]. At present, the reports on microbial degradation of CY were relatively small, so was the research on the CY degrading strains. There were some studies the characteristics of CY-degrading Strain. Chen et al. [70,128] isolated a strain of degrading bacterium streptomyces sp. HP-S-01 from the chemical plant waste-water treatment pool of long-term production of pyrethroid pesticides, and the streptomyces could completely degrade CY by using Andrews equation of streptomyces HP-S-01 to analyze the dynamic process of degrading CY, besides, further physiological characteristics of degrading bacteria was studied. First, there was a relative curve of Streptomyces HP-S-01 growth and the degradation of CY in the result. It can be seen from the Figure 2, CY’s degradation and cell growth were positively correlated with the existence of CY. In the presence of CY, streptomyces’ growth had no obvious retention period, and it quickly entered the logarithmic growth phase, and logarithmic growth phase of streptomyces was one to two days. In the process, the degradation to β-CY in the Streptomyces was the fastest. Second, there was the influence of bacterial count on streptococcus HP-S-01 to degrade CY. When the amount of inoculation in streptomyces was 0.2–0.6 g/L, the degradable rate showed an upward trend in total. From that, the visibly degradable ability of streptomyces can improve in a certain range with the increase of the amount of inoculation. When the inoculated amount was up to 0.6 g/L, the degradable rate rose rapidly, then the concentration had the greatest influences on the degradation of CY. But, when the inoculated amount was higher, there was no effect. Third, there was the effect of temperature on streptomyces HP-S-01 to degrade CY. Obviously, the best degradable temperature was 28 °C, higher than the temperature or below this temperature, the degradable effect would be inhibited, and the degradable rate would decrease. Especially in 18 °C and 38 °C, the degradable ability was the lowest. Finally, pH affected the degradation of CY by streptomyces sp. HP-S-01. When pH < 7.5, the streptomyces’ degradation of β-CY ratio increased with the increase of pH, when pH > 7.5, streptomyces’ degradation of β-CY decreased with the increase of pH, which indicated that the most suitable pH was 7.5. It also showed that the bacteria had better degradable ability under partial alkaline conditions.

Singh [5] compared the Chabishi medium, potato dextrose agar, Richard medium, oatmeal medium and corn powder liquid medium to study the effect of five kinds of medium on CY degrading strain Fusarium sp. HG-P-01 growth, and the result showed that the Chabishi medium was the most suitable medium for mycelial growth. Through this experiment, the optimum concentration of formula, carbon nitrogen, and phosphorus and the expected degradable rate were established. There was a sample of CY HPLC chromatogram. CY standard sample’s peak was composed of two peaks, and the peaks were sharp, symmetry, stable, and the reserved time were 3.643 min and 3.840 min. The standard curve equation was Y = 51.309X + 10.932, and the correlated coefficient was R^2^ = 0.9999. The concentration of reagent was significantly relative with corresponding peak area, so the results were reliable. Tallur et al. [129] studied the synergistic effect of mixed microbial to degrade CY by field’s tests, and determined the effects of different temperatures, different spraying time, different degradable bacteria concentration, and different days on the degradable rate of CY. In the result, the optimum water bath temperature was 33 °C, the spraying time was 5 pm., the concentration of cell was OD_600_ 1.0. Zhang et al. [79] isolated and screened two strains of Serratia spp. strain JC1 and JCN13 from activated sludge, and then studied the degradable ability to the CY and the cell’s surface hydrophobicity. The results showed that JCN13 has higher hydrophobicity and degradation ability than JC1, which mean that the high hydrophobicity of the cell of degrading bacteria can enhance the degradation of CY. There was a comparison of cell’s surface hydrophobicity of strain L12 in xylene and n-octanol [79]. The hydrophobicity of bacterial cell’s surface was one of the most important determinants of nonspecific bacterial adhesion of bacteria to variously biological and abiotic surfaces and interfaces, and it was also one of the main elements that affected bacterial uptake and the degradation of hydrophobic organic matter [93]. It had important significance of studying the cell’s surface hydrophobicity to degrade organic compounds in non-aqueous phase.

## 5. Microbial Degradation of 3-phenoxy Benzoic Acid (3-PBA) and its Application

### 5.1. Structure and Properties of 3-PBA

3-PBA was white powder crystal, molecular formula C_13_H_10_O_3_, molecular weight is 214.2, and the melting point of 149–150 °C. It was difficult to dissolve in water (20 °C water solubility is 200 mg/L) and is easy to dissolve in organic solvents. 3-PBA was one of the degradable products of most CY pesticides [130]. Some researchers showed specific esterase produced by bacteria acted on the ester bond in the molecule of CY pesticides. In the case of CY, it became two intermediates contained lnulin component and cyanalcohol [130,131,132]. Alpha α-cyano-3-benzyl alcohol produced in the process that could be further oxidized to 3-PBA [130,131]. Deng et al. found that the metabolites of the process of β-CY degradation was 3-PBA by using the method of HPLC chromatogram, that was to say that the β-CY could be degraded to 3-PBA. After that, the metabolites of 3-PBA’s degradation by strain YAT was further studied [133].

### 5.2. Current Problems of 3-PBA

3-PBA was one of the most degradable intermediates to pyrethroid pesticides, and had certain estrogenic properties, the faster migrated rate, longer half-life and stronger reproductive toxicity [113,134,135,136,137,138]. When comparing with pyrethroid pesticide, its hydrophobicity was relatively weak, and it was easier to migrate and accumulate in the environment. More and more 3-PBA remained in the soil, agricultural, livestock products, and even the body, did harm to the ecological environment and human’s health and had more hazard potential effect than pyrethroids because its antibacterial activity restricted the bio-degradation of β-CY [130]. 3-PBA produced secondary pollution in the environment and agriculture, at the same time, it also blocked the pyrethroid pesticide’s degradation into small non-toxic molecules [139], making the pesticide’s residue become more serious. Therefore, the degradation of 3-PBA was the key to the elimination of pyrethroid pesticide pollution [140]. How to reduce and eliminate the pollution of 3-PBA in the environment and agricultural products had received extensive attention [139,140], but the utilization of biodegradable enzyme was thought to be the effective way to reduce or eliminate the pesticide residues in agricultural products and control the environmental pollution by pesticide [134,135,137]. At present, it was reported that active sludge and soil were the main screening sources of 3-PBA degrading bacteria [78,132,136].

### 5.3. Degrading Bacteria of 3-PBA and Simultaneous Degradation of 3-PBA and CY

At present, the degrading microbes of 3-PBA were mainly Pseudomonas, fungi and actinomycetes were relatively few, and at this stage, studies of bio-degradation of 3-PBA remained at the screening and degrading bacteria [141]. The strains have been proved to be the following strains, for example, Sphingomonas sp.SC-1 from the active sludge [133], Aspergillus niger YAT1 in brick tea, the Eurotium cristatum ET1 in Fuzhuan tea [113], Aspergillus oryzae M-4, Sphingomon As sp.JZ-2, and Micrococus sp. CPN1 in soy sauce koji [142], etc. SC-1 could completely degrade 300ug/mL in one day, which was currently reported to be able to degrade 3-PBA and as the sole source of carbon mineralization to degrade it [133]. On the other hand, Deng et al. obtained a set of data by using HPLC–UV method to screen degrading bacteria of β-CY and 3-PBA, which could be seen in Table 4 [81]. Synergy was an important way to degrade pesticide’s residues by bacteria. What has already been mentioned above could improve the degradable rate of pyrethroid pesticides, but few reported to degrade β-CY and 3-PBA on the same time. Dg-s-01 could endure and degrade high concentration of 3-PBA (100 mg/L) and degrade β-CY by using sphingobium at the same time [81]. Deng et al. established a HPLC method to determine the concentrations of both β-CY and 3-PBA simultaneously in degradable systems. They found a novel β-CY degrading strain, Bacillus licheniformis B-1 isolated from the soil of tea garden, utilized β-CY as growth’s substrate. First, they identified the detector wavelength of β-CY and 3-PBA. The baseline was in fact improved at 210.00 nm and high-resolution peaks received at this wavelength were narrow and symmetric, the wavelength of 210 nm, and the proximity of the maximum wavelengths, was deemed to be provided for the simultaneous determination of both β-CY and 3-PBA. The intra-day’s and inter-day’s peak areas of β-CY and 3-PBA were compared; recovery also was tested. The limits of detection were 0.06 and 0.13 µg/mL for β-CY and 3-PBA respectively, and the corresponding limits of quantification were 0.21 and 0.34 µg/mL respectively. Spiking recoveries for β-CY varied from 98.38% to 105.80%, with relative standard deviations (RSDs) varying from 1.49% to 3.93%. Spiking recoveries for 3-PBA varied from 99.59% to 101.20%, with RSDs varying from 0.58% to 3.64% [113]. Deng et al. studied the kinetic parameters of degradation of 3-PBA by YAT under the effects of different factors, and the results are shown in Table 5 [81]. The results showed that alkaline conditions could increase the 3-PBA’s degradable rate, whereas the acidic conditions might increase the stability of 3-PBA and its resistance to chemical and microbial degradation of 3-PBA. Except that, they also discussed the growth’s characteristics and the degradable capacity of 3-PBA for Aspergillus niger YAT. The results showed that 3-PBA made some differences in the growth of YAT; it not only prolonged the lag phase of YAT, but also affects its final amount of growth. The strain could degrade all of the 3-PBA (100 mg/L) in 22 h, which proved that YAT was highly efficient [81]. The two bacteria PBM11 and CDT3 played a role synchronously in the degradation of CY and 3-PBA, which accelerated the degradable rate of CY and reduced pesticide residues. But, during culturing the CY and strain PBM11 alone, the existence of CY on the growth of strain PBM11 had no obvious effect. Similarly putting degrading bacteria CDT3 and 3-PBA alone, the presence of 3-PBA could produce a certain inhibition on degrading CY and the degradable rate was negatively correlated with the concentration of 3-PBA. But, when the concentration of 3-PBA was lower than 200 mg/L, the final degradable rate of CDT3 to CY was unchanged after a sufficiently long time. In addition, the study also showed the optimum proportion of strains CDT3 and PBM11 to degrade CY and 3-PBA.

## 6. Conclusions and Outlook

The microbial degradation studies of pesticides had been greatly developed, and most of the pesticide degrading microbial strains have also been identified, but the actual application of microbial bioremediation was limited, which was often due to its low-degradable efficiency and the environment condition. Mineralization and co-metabolism were the main mechanisms for the further degradation of pesticides and their intermediate products, while the group and molecular structure of pesticide determined its degradation behavior in the microbial environment, chemical structure determined its solubility, in which molecular orientation, spatial structure, chemical functional groups, intermolecular attraction, and repulsion characteristics effecting the ingestion of pesticides by microorganism. The main research directions of microbial degradation of pesticides were: the development of high efficiency pesticide degradation engineering bacteria, the cultivation of mixed bacteria, the immobilization of degrading bacteria, the research of pesticides-degrading fungi, and the quantitative study of pesticide biodegradation model. In recent years, with the development of genetic engineering and molecular biology, on the one hand, researchers began to shift to the construction of efficient engineering bacteria, and used the gene recombination technique. On the other hand, they transformed enzyme gene to construct the vector that could express efficiently the characteristics of degrading pesticide. After that, engineering bacteria could be received. The purpose of that was to improve the expression level of specific proteins or enzymes, so as to improve the efficiency of degradation, which could overcome the problem that some enzymes in the environment could not be stabilized and maintain a high enzyme activity. In short, there was an effective method to eliminate pesticide pollution, which was using microbial agents or fertilizer preparation applied in polluted environment. The difference of pyrethroid degradation in the human body was still not very clear. Although the isolation and screening of degrading bacteria and their degradable effect were better, the research of synergistic degradation target of various degrading bacteria was rarely seen. What was more, a large number of experimental studies could not be applied in practical production. A large number of 3-PBA residues not only caused two pollutions of agricultural products, but also led to pyrethroid pesticides being blocked in biomineralization, which indirectly caused the pesticide residue problems to become more serious and had a threat to food safety, the environment, and human health. The isolation and screening on degradable strain of 3-PBA and the degradable characteristics of strains have been studied currently, but there was no related literature and reports that combined specific methods to study the degradation mechanism of 3PBA being degraded to phenolic compounds. For example, chromatography and mass spectrometry, degradable pathway, catalytic mechanism, enzymatic characteristics, and so on should be emphasized. Although heterologous compounds may be partially or completely decomposed by some microorganisms, they may be resistant to degradation in the environment due to their greater structure, insolubility, and high thermal stability. Therefore, we also need to pay attention to this problem to ensure strain and xenobiotic compounds’ degradation products harmless in pollution remediation [143,144].

## Figures and Tables

**Table 1:** Kinds of pesticides used in agricultural production.

Types of Pesticides		Name of Pesticide
Insecticide	Organic nitrogen	Benzoylphenyl Ureas [29], chlordimeform [30].
	Organic phosphorus	Acephate [31], azinphos-methyl [32], bromophos [32], chlorpyrifos [31,32], coumaphos [33,34], diazinon [31,32,33], dimethoate [18,31,33,34],dioxathion [34], disulfoton [32,33], diazinon [32,33], ectophos [34], fenitrothion [31,32], fenitrooxon [32], fonofos [32], glyphosate [32,33], leptophos [33], malathion [31,32,33,34,35], mathamidophos [33], parathion [32], phenthoate [31,33], profenofos [33], phorate [33], phosmet [36], phosphothion [34], trichloffon [34], trichlorfon [33]
	Organic chlorine	Aldrin [18,32,35], chlordane [32,35,37], DDT [32,35], dieldrin [32,35], dicofol [31], endosulfan [31,32,35], endrin [32], fipronil [31], heptachlor, [32,35], lindane [35,38], γ- BHC [34], γ- hexachlorocyclohexane [38]
	Carbamate	Aldicarb [39], carbaryl [31,34], carbofuran [31], carbosulfan, [31], cartap [31]
	Pyrethroid	Cypermethrin [31], chlorfenvinphos [31], deltamethrin [31], fenvalerate [29], flumethrin [31], permethrin [31], ivermectin [31]
	Insect growth regulators	Azadirachtin [40], benzoylphenylurea [40], diflubenzuron [40], methoxyfenozide [40], pyriproxyfen [40], spinosad [40], tebufenozide [40]
Acaricides	Amitraz [41], coumaphos [21,41], dimethoatet [18], fenpyroximate [41], formic acid [41], menthol [41], tau-fluvalinate [41], thymol [41]	
Herbicide	Acetanilides [42], alachlor [39], barban [35,43], chlorbromuron [35], hlorophenoxy [42], dalapon [35], diuron [35,44], glyphosate [45], linuron [35,46], monuron [36], neburon [36], pendimethalin [36], pentachlorophenol [36,47], propham [35], salted iron phosphorus [45], swep [35], 2,4-D [48], 2,4,5-T [35]	
Bactericide	Bayleton [48], blue copper [48], chlorothalonil [43], copper hydrochloride [48], copper oxychloride [48], copper sulphate [48], different rice blast net [45], dithane [48], dithiocarbamates [42], mancozeb [48], metalaxyl [45,49], methyl phosphorus [45], impact [45,48], polytrin [48], ridomil [48], rice blast net [45], triazoles [42], thiocarbamates [42], thiovit [48]	

**Table 2:** Commonly pesticide degrading microorganism.

Types of Microorganism	Species	Example of Pesticide Degradation
Bacteria	Pseudomonas	Aldrin [20], chlorpyrifos [20], coumaphos [33], ddt [20], diazinon [20,33], endosulfan [20], endrin [20], hexachlorocyclohexane [20], methyl parathion [20,33], monocrotophos [20], parathion [20,33]
	Bacillus	Chlorpyrifos [20,33], coumaphos [33], DDT [20], diazinon [20], dieldrin [20], endosulfan [20], endrin [20], glyphosate [20,33], methyl parathion [20,33], monocrotophos [20], parathion [20,33], polycyclic aromatic hydrocarbons [20]
	Alcaligenes	Chlorpyrifos [20], endosulfan [20,52]
	Flavobacterium	Diazinon [33], glyphosate [33], methyl parathion [33], parathion [33]
Actinomycetes	Micromonospora, Actinomyces, Nocardia, Streptomyces	Aldrin [20], carbofuran [53], chlorpyrifos [20,56], diazinon [56], diuron [44]
Fungus	White rot fungi, Rhizopus, Cladosporium, Aspergillus fumigatus, Penicillium, Aspergillus, Fusarium, Mucor, Trichoderma spp, Mortierella sp.	Alachlor [39], aldicarb [39], atrazine [39,54], carbofuran [35], chlordane [35], chlorpyrifos [33], DDT [35], diuron [57], endosulfan [32,58,59,60], esfenvalerate [61], fenitrothion [62], fenitrooxon [62], fipronil [63], heptachlor epoxide [64], lindane [35,38], malathion [35] metalaxyl [49], pentachlorophenol [35], terbuthylazine [57], 2,4-D [35]
Algae	Small green algae	Phorate [45], parathion [45]
	Chlamydomonas	Atrazine [55], fenvalerate [65]
	Genus of diatoms	DDT [66], patoran [66]

Description: Bacteria has strong adaptability and is easy to induce mutations and occupies the main position in the study of degradation of pesticide. In addition to the above, there are a lot of pesticide degrading bacteria, such as Escherichia coli, Clostridium, Escherichia coli, Bacillus licheniformis, Thiobacillus and so on.

**Table 3:** Kinetic parameters of degradation of β-CY by YAT under the effects of different factors (Taken from Deng et al. 2015).

Factors		Kinetic Equation	T_1/2_ (Day)	K_d_ (mg/(L·day)^−1^)	R^2^
	25	C_t_ = 25.78e^−0.194t^	3.573	0.194	0.924
C_0_ (mg/L)	50	C_t_ = 50.14e^−0.110t^	6.301	0.110	0.921
	100	C_t_ =100.81e^−0.059t^	11.748	0.059	0.950
	25	C_t_ = 50.25e^−0.103t^	6.730	0.103	0.945
Temperature (°C)	30	C_t_ = 50.14e^−0.110t^	6.301	0.110	0.921
	35	C_t_ = 50.21e^−0.118t^	5.874	0.118	0.990
	6.0	C_t_ = 50.37e^−0.094t^	7.374	0.094	0.910
pH	7.0	C_t_ = 50.14e^−0.110t^	6.301	0.110	0.921
	8.0	C_t_ = 50.09e^−0.120t^	5.776	0.120	0.915

**Table 4:** Degradation characteristics of the β-CY and 3-PBA-degrading bacteria.

Strain	Accession Number (NCBI)	Source	Degradation Characteristics (%)	
			β-CY	3-PBA
B. licheniformis B-1	HQ009796	Tea garden soil	52.91%	—
Aspergillus oryzae M-4	JF461319	Soy sauce koji	26.01%	80.10%
Sphingomonas sp SC-1	JN857975	The sludge of pesticide factory wastewater	—	99.99%

Note: ‘—’means no degradation.

**Table 5:** Kinetic parameters of degradation of 3-PBA by YAT under the effects of different factors (Taken from Deng et al. 2015).

Factors		Kinetic Equation	T_1/2_ (h)	K_d_ (mg/(L·h)^−1^)	R^2^
	50	C_t_ = 50.22e^−0.123t^	5.635	0.123	0.935
C_0_ (mg/L)	100	C_t_ = 101.24e^−0.101t^	6.863	0.101	0.924
	150	C_t_ = 150.51e^−0.057t^	12.160	0.057	0.957
	25	C_t_ = 100.87e^−0.062t^	11.180	0.062	0.943
Temperature (°C)	30	C_t_ = 101.24e^−0.101t^	6.863	0.101	0.924
	35	C_t_ = 100.41e^−0.107t^	6.478	0.107	0.963
	6.0	C_t_ = 100.87e^−0.093t^	7.453	0.093	0.902
pH	7.0	C_t_ = 101.24e^−0.101t^	6.863	0.101	0.924
	8.0	C_t_ = 100.97e^−0.106t^	6.539	0.106	0.970
